# Prevalence of histological findings of human papillomavirus (HPV) in oral and oropharyngeal squamous cell carcinoma biopsies: preliminary study

**DOI:** 10.1016/S1808-8694(15)31208-8

**Published:** 2015-10-20

**Authors:** Sandra Doria Xavier, Ivo Bussoloti Filho, Carmem Lúcia Penteado Lancellotti

**Affiliations:** 1 Otorhinolaryngologist. Master degree.; 2 Adjunct Professor of Otorhinolaryngology, PhD- Santa Casa de Misericórdia de São Paulo.; 3 Adjunct Professor, PhD in Pathology – Pathology Department - Faculdade de Ciências Médicas da Santa Casa de Misericórdia de São Paulo. Irmandade da Santa Casa de Misericórdia de São Paulo.

**Keywords:** carcinoma, papillomavirus, mouth, oropharynx

## Abstract

Human papillomavirus (HPV) is considered to be an etiologic agent of cervical cancer and, recently its relation to oral and oropharyngeal cancer has also been investigated. Oral squamous cell carcinoma (SCC) represents 90% of all malignant tumors that affect the oral cavity. The prevalence of HPV in patients with SCC ranges from 0 to 100%. The most known viral cytopathic effect is koilocytosis, considered to be a major characteristic of HPV infection. **Aim:** The aim of this study was to verify the prevalence of some peculiar characteristics of HPV - koilocytosis - in oral and oropharyngeal SCC. **Study design:** transversal cohort. **Material and method:** Twenty slides with oral and/or oropharyngeal SCC were examined under microscopy. **Results:** in 15 of them, koilocytosis was found, amounting to 75%. Although we know that polymerase chain reaction (PCR) is the method with the best sensitivity for HPV detection, we began this research looking for koilocytosis, which is highly suggestive of HPV infection. **Conclusion:** This study is a trial project and we will continue this research with PCR measures to confirm this high prevalence of HPV infection in oral and oropharyngeal SCC.

## INTRODUCTION

The Human papillomavirus virus (HPV) is a small sized virus of intranuclear replication with diameter of approximately 50 nm ^1^. It may infect mucosa and skin surfaces in almost all vertebrate species^2^. Seventy types of HPV ^3^ have been identified so far and were classified according to its distribution on nucleic acids in the viral genome. The subtypes found in the oral cavity were 1, 2, 4, 6, 11, 13, 16, 18, 30, 31, 32 and 57^4^.

Comprehensive research studies related some subtypes of HPV to malignant and pre-malignant lesions of cervix, vulva, penis, conjunctive and upper digestive tract and airways^5^. HPV is universally accepted as an etiologic agent of cervical cancer, and its relation to oral and oropharynx cancer has recently been investigated. The most popular viral cytopathic effect of HPV is koilocytosis and is considered a “major criterion” in HPV infection from the pathophysiological point of view. Koilocytotic atypia consists in nuclear atypia and perinuclear vacuolization. ^6^

Today, there are molecular biology methods to study HPV such as in situ hybridization (ISH), hybrid capture and PCR that provide not only increased sensitivity in diagnosis, but also the typing of HPV, but costs of such exams are higher if compared against the detection of koilocytosis hematoxylin-eosin stained slides.

### HPV and Oral Spinocellular Carcinoma (OSC)

Oral squamous cell carcinoma is also known as epidermoid or oral spinocellular carcinoma (OSC) and accounts for 90% of all malignant tumors that affect the oral cavity^7^. The most common factors associated with oral cancer development are smoking, alcohol, syphilis, nutritional deficiencies, sunray exposure, trauma, poor hygiene and irritation of the teeth or dental prosthetics^8^. In addition to those factors, the viruses have been investigated as likely carcinogenic agents. Sÿrjanen et al. ^9^ suggested HPV may play a role in oral cancer if associated with cell abnormalities found in malignant and pre-malignant lesions of the mouth similar to those that occurred in cervical cancer. The HPV infection is identified as the most common cause of 95% of cervical carcinomas and a large rate of other genital carcinomas^10^.

Tobacco and alcohol abuse are well established risk factors for oral cancer, but a small rate (15-20%) of the patients do not have any past history of smoking or alcohol addiction suggesting the presence of other risk factors ^11^ such as HPV, but its role has not been clearly defined yet. An intriguing finding is the rate of approximately 40% reduction in death risk in patients with positive HPV tumors.

HPV 16 is the most common type of virus associated with oral and cervical cancer 12, 13 whereas types 6 and 11 are more commonly found in benign and pre-malignant lesions and rarely found in neoplastic head and neck lesions^14^.

The importance of HPV infection in oral carcinogenesis is supported by the ability of high risk HPV to immortalize oral keratinocytes*in vitro* ^15^.

## OBJECTIVE

The objective of this study is to determine the prevalence of suggestive findings of HPV, koilocytosis in OSC of the oral cavity and oropharynx through histological analysis of such lesions.

## MATERIAL AND METHOD

Twenty patients diagnosed with OSC of the oral cavity and oropharynx were examined with optical microscopy to investigate suggestive signs of HPV infection in hematoxylin-eosin stained slides.

Concomitantly, the records of the 20 cases were investigated as shown in Table 1.1.

**Table 1:** List of patients with oral or oropharynx OSC.

Initials	Age	Gender	Race	Smoking	Alcohol abuse	Site	Staging	Lesion	C
F.V.S.	86	M	B	?	?	JugalMucosa	?	Ulcer	-
A.S.	69	M	B	-	-	Hard palate	T4N1Mx	Ulcerated infiltrating	-
M.H.M.	42	M	P	-	-	Hard palate	T4NoMo	Ulcerated infiltrating	-
D.C.	51	M	B	+	-	Floor	?	Infiltrating	-
C.A.S.	61	M	B	+	-	Soft palate	T1NoMo	Ulcer	-
I.D.	48	M	B	-	-	Gums	?	Bone reabsorption	+
C.M.F.	55	M	B	?	?	Soft palate	T4N2cMx	Ulcerated infiltrating	+
J.Z.T.	58	M	B	+	+	Floor	?	Ulcerated infiltrating	+
R.A.S.	71	M	B	?	?	Floor	?	Infiltrating	+
A.C.P.	63	M	B	+	+	Oropharynx and larynx	T4N2cMx	Infiltrating ulcerated	+
G.A.Z.	47	M	B	+	+	Soft palate	TxN3Mx	Infiltrating ulcerated	+
J.C.G.M	63	M	B	+	+	Soft palate	?	Infiltrating ulcerated	+
T.C.M.	56	M	B	?	?	Oropharynx	T4N2Mo	Infiltrating ulcerated	+
S.B.C.	56	M	B	?	?	Hard palate	?	Infiltrating ulcerated	+
J.T.S.	80	M	N	+	+	Tongue	T3N1Mx	Infiltrating ulcerated	+
M.E.V.	55	F	P	+	-	Tongue	T4N1Mx	Infiltrating ulcerated	+
R.A.A.	64	M	N	+	-	Oropharynx and base of tongue	T4N2cMx	Vegetating	+
R.D.O.	53	M	B	+	-	Oropharynx	T4NoMo	?	+
A.G.V.	53	M	B	+	+	Floor	T4N2cMo	Exophytic ulcerated	+
E.F.F.	47	M	P	?	?	Tongue and Floor	T4N2cMo	Infiltrating ulcerated	+

C: koilocytosis

T: tumor

N: lymph node

M: metastasis (TNM classification)

?: non-reported in patient’s records

M: male

F: female

B: white

P: brown

N: black

+: present

-: absent

## RESULTS

Seventy-five percent of the cases (15/20) presented koilocytosis, and were identified as positive cases of koilocytosis (C+).

The survey in patients’ records showed that 19 patients were male and 1 patient was female, ages ranging from 42 to 86 years (mean age= 58.9). Fifteen patients were White, three were Brown and two were Black. The group of C+ patients had ages that ranged from 47 to 80 years (mean age= 57.9). The group of C-patients had ages that ranged from 42 to 86 years (mean age= 61.8).

Eleven patients were smokers (55%) and six were alcohol abusers (30%), with 6 patients (30%) - 5 C+ and 1 patient C- that did not have any smoking or alcohol history in their records. The group of C+ patients had 60 % of smokers and 40% of alcoholics, whereas the group C- had 40% smokers and did not have any alcoholic patient as shown in Table 2.

**Table 2:** Smoking and alcoholism in patients with positive and negative koilocytosis.

	C +(n = 15)	C -(n = 5)
Smoking (n = 11)	60%	40%
Alcoholism (n = 6)	40%	0%
+ Common Site	Palate (27%) and floor (27%)	Palate (60%)

C+: koilocytosis

The tumor location varied. Most occurred in the palate (35%) - 20% in the soft palate and 15% in the hard palate – followed by the oral cavity floor (25%), oropharynx (20%), tongue (10%), gums (5%) and jugal mucosa (5%). In the C+ group, the most common sites were palate and oral cavity floor, both with 27% rate, whereas in the group C- the most common site involved was the palate region (60%).

In regards to the clinical aspect upon diagnosis, most of the patients had ulcerated infiltrating lesion (60%) and in advanced staging - T4 (50%), in 7 patients (35%) - 5 C+ and 512 2 C- - did not have any report of clinical staging in their records. In the C+ group, 66% of the patients had ulcerated infiltrating lesion and 54% were in advanced staging, whereas in the C- group, 40% of the patients had ulcerated infiltrating lesions and 40% were in advanced staging.

## DISCUSSION

In this study, koilocytosis was found in 15 out of the 20 slides investigated, accounting for 75% of the cases.

**Figure 1:**
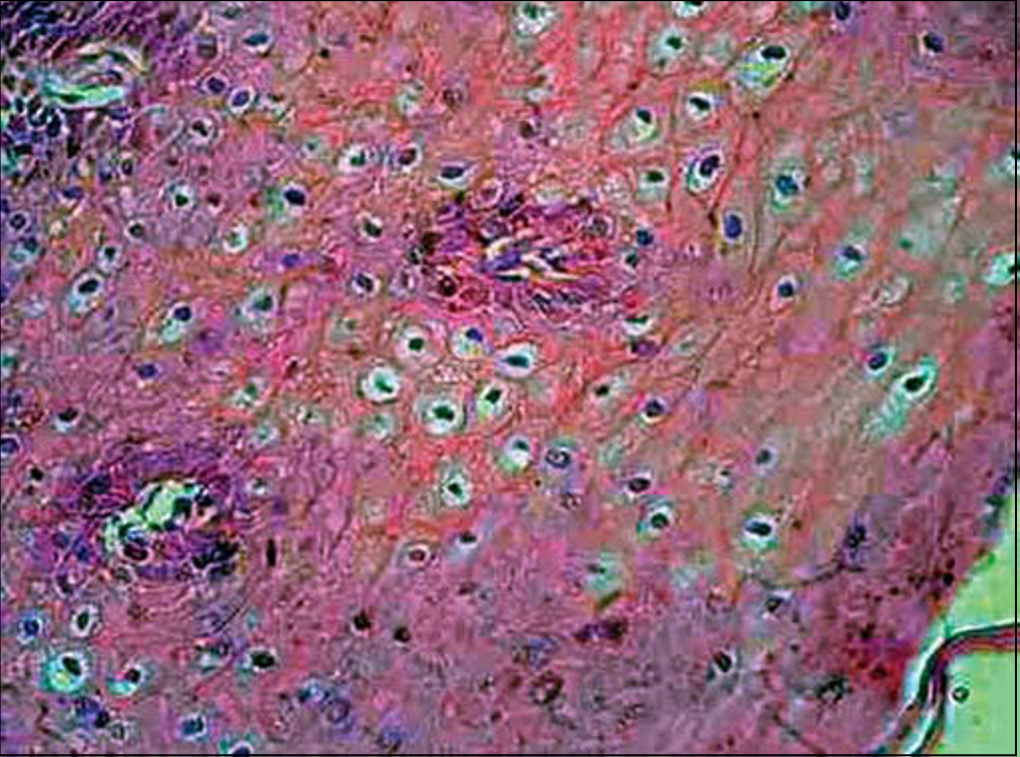
Hematoxylin-eosin stained slides showing koilocytosis: cells with picnotic halo, surrounded by clear outside halos with volume generally higher than that of the cytoplasm.

Koilocytosis first described by Leopoldo Koss et al. in 1956^16^, consists in a picnotic nuclei surrounded by extensive clear halos with a volume generally higher than the cytoplasm if observed in slides under optical microscope, as seen in Figure 1.

According to some authors 17, 18, the koilocytosis is a pathognomonic sign of HPV infection serving as a foundation for molecular biology studies.

Some papers in the literature report that HPV frequency in oral carcinoma ranges from 0 to 100%^13^, ^19^whereas other investigators report it ranges from 18 to 100% 12, 14. The mean frequency of 25% was estimated by Garlick^20^ in his review about the topic. As it can be seen, the variability is extreme and may be a consequence of different sample sizes or methods used with variable sensitivity and specificity.

Herrero et al. ^21^ demonstrated HPV DNA in 3.9% of 766 cancer biopsies of the oral cavity and in 18.3% of the 142 biopsies of oropharynx cancer, through the PCR study. Klussmann et al. ^22^ found 18% of HPV DNA in cancer biopsies of the oral cavity, 8% in nasopharynx cancer, 25% in hypopharynx cancer and 45% in oropharynx cancer and particularly in tonsil carcinomas (58%). Ritchie et al. ^23^ detected HPV in 21% of the oral cavity and oropharynx cancers, with 83% of HPV -16. Ha et al. ^24^ reported 0.98% of HPV DNA in pre-malignant lesions and only 2.9% in malignant lesions of the oral cavity.

Niv et al. ^25^ conducted a study to investigate HPV presence in oral cavity carcinoma and found a positive rate of 17.3%; in all cases the tumor was located in the anterior tonsillar pillar. In other studies, the most common tumor sites where they found HPV were jugal and palate mucosa (40-50%) ^26^. In this study, the most common locations were palate and oral cavity floor, both with 27% prevalence.

Positive HPV carcinoma seem to be a distinctive entity (involve more basal cells and have lower inflammatory component), with distinct biology (less p 53 mutation), distinct risk factors (less association with tobacco and alcohol and a different clinical outcome - higher survival rate) ^11^. Conversely, this study did not show lower association with tobacco and alcoholism, but higher percentage of alcoholic and smokers in group C+.

Cruz et al. ^27^ observed that patients under 60 years of age with oral carcinoma presented eight times higher risk of being infected by HPV than people older than 60 years. In this study, men presented increased HPV positive responses than women, but an association between HPV infection and tumor location, clinical staging in diagnosis or smoking, and alcoholism could not be found. This survey did not show any significant difference of age range between groups C+ and C-.

Klussmann ET al. ^28^ reported a statistically significant difference between HPV prevalence in oropharynx tumor (45%), especially in palatine tonsils (58%) against the other sites of the oral cavity (7 to 25%).

Some authors 11, 28 believe that positive HPV palatine tonsil carcinomas constitute an independent tumor entity. Klussmann et al. ^28^ found out that these patients present statistically lower exposure level to known risk factors such as smoking and alcoholism. They also found a statistically significant correlation between HPV and low level of tumor differentiation, contrary to the previous findings the authors mentioned.

Miller and Johnstone^29^ concluded in their meta-analysis from 1982-1997 that HPV is an independent risk factor and extremely important in OSC, and their results indicated that HPV detection is twice to three times more common in oral pre-malignant lesions and 4 to 7 times more common in OSC if compared against normal oral mucosa.

Brandwein et al. ^30^ did not find any statistical association between HPV and TNM staging and also between clinical and differentiation aspects of the tumor. In this study, there were no statistically significant differences found between negative and positive HPV regarding its disease-free interval and survival time.

Although we know that PCR is the best current method to detect HPV this study began by investigating koilocytosis in hematoxylin-eosin stained slides, which is highly suggestive of HPV infection. This study is part of a pilot project that will be followed up by PCR tests to be performed in all slides previously examined to confirm the high prevalence of HPV infection in oral OSC. The histological analysis by itself may suggest the presence of HPV and is a screening method of HPV-related lesions. It is quite a useful method in centers in which sophisticated molecular biology survey methods are unavailable.

## CONCLUSION

Careful anatomic pathological survey of OSC of oral cavity and oropharynx showed high prevalence of koilocytosis - 75% -, indicating a likely high HPV prevalence in this type of tumors.
